# Driving mesenchymal stem cell differentiation from self-assembled monolayers

**DOI:** 10.1039/c7ra12234a

**Published:** 2018-02-09

**Authors:** L. S. Tew, J. Y. Ching, S. H. Ngalim, Y. L. Khung

**Affiliations:** Regenerative Medicine Cluster, Advanced Medical and Dental Institute (AMDI), Universiti Sains Malaysia 13200 Kepala Batas Pulau Pinang Malaysia; Institute of Biological Science and Technology, China Medical University No. 91 Hsueh-Shih Road Taichung Taiwan 40402 Republic of China; Institute of New Drug Development, China Medical University No. 91 Hsueh-Shih Road Taichung Taiwan 40402 Republic of China Yitlung.khung@mail.cmu.edu.tw

## Abstract

The utilization of self-assembled monolayer (SAM) systems to direct Mesenchymal Stem Cell (MSC) differentiation has been covered in the literature for years, but finding a general consensus pertaining to its exact role over the differentiation of stem cells had been rather challenging. Although there are numerous reports on surface functional moieties activating and inducing differentiation, the results are often different between reports due to the varying surface conditions, such as topography or surface tension. Herein, in view of the complexity of the subject matter, we have sought to catalogue the recent developments around some of the more common functional groups on predominantly hard surfaces and how these chemical groups may influence the overall outcome of the mesenchymal stem cells (MSC) differentiation so as to better establish a clearer underlying relationship between stem cells and their base substratum interactions.

## Abstract 2

Graphical illustration showing the functional groups that drive MSC differentiation without soluble bioactive cues within the first 14 days.

## Graphical abstract

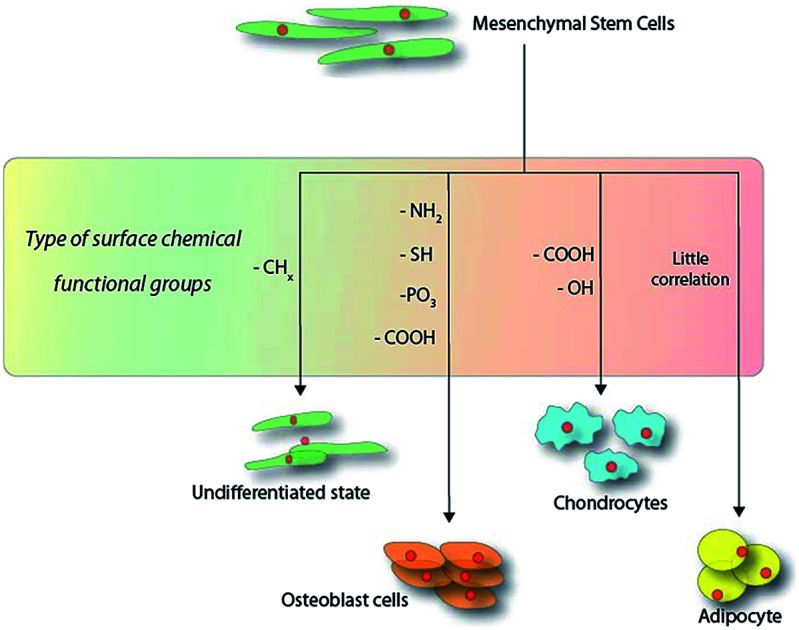

## 1. Introduction

In view of the advancements made in biomaterial technology in recent years, having a bioactive surface had already been deemed as a necessary prerequisite towards promoting cellular longevity and biofunctionality. Hence, many biomaterials have been designed presenting a bioactive surface towards the incoming adhering cells to attain desired outcomes. As biomaterial-based systems have already found wide applications in many areas, such as cardiovascular,^1–3^ ophthalmologic,^4–6^ biosensors^7–9^ as well as drug delivery systems,^10–13^ it is certainly inevitable that these platforms would also attract notable attention in stem cell differentiation studies. In fact, well-customized biomaterials have already been shown to possess the ability to restore the physiological functions of devastated tissues or organs caused by disease, trauma or natural ageing *via* their potential regeneration properties.^14,15^ In principle, the selection criteria of biomaterials for tissue regeneration and artificial implantation would encompass many overlapping aspects, such as surface biocompatibility, bioactive, bioinert, biodegradable, bioresorbable and mechanical strength. However, no single biomaterial candidate could fully claim to harness all the above characteristics at the same time. Thus, in many situations, it is necessary to redesign a “smart material's” surface composition so as to better control the interactions between biomaterials and living tissues and ultimately to optimize their therapeutic functions.

Directly conjugating signalling molecules, such as growth factors and differentiation factors, on surfaces for stimulating cell migration, growth and differentiation has been widely investigated in recent years.^16–19^ Growth factors, such as vascular endothelial growth factor (VEGF), transforming growth factor-beta (TGF-β), fibroblast growth factor (FGF), platelet-derived growth factor (PDGF) and insulin-like growth factor (IGF), are some of the most common biocues that have been frequently immobilized onto a substratum in order to promote cell differentiation.^20–25^ Despite the fact that signalling molecules may provide all the necessary instructive signals to promote the differentiation of stem cells, heterogeneous populations of cells are often observed at various stages in the differentiation timeline. This divisive outcome may present itself as a major challenge in long-term tissue engineering.^26,27^ Moreover, pleiotropic effects could occur from the simultaneous activation of multiple intracellular signalling pathways^28–30^ and could sometimes lead to some less than desired outcomes. Furthermore, they may also trigger other pathological responses, such as inflammation, neurogenesis, endocrine function, haematopoiesis.^31^ Some of the properties of biomaterial surfaces with effects on the biological response of cellular behaviour have been reviewed lately.^32^

In actuality, the literature has almost been overwhelmed by many interesting hybrid systems reported (chemistry + topography, *etc.*) for the differentiation of stem cells, where each of these systems offer individual merits. For instances, over the past decade, Whitesides and co-workers strongly advocated the use of highly regulated SAMs in parallel with surface topography as a means to direct the stem cell fate through a biochemical response of cells^33,34^ towards these surface cues. They argued that a model of mixed SAMs that accounts for homotypic stem cells interaction and cell surface interaction would support the stem cell differentiation. These mixed SAMs systems could modulate many of the surface parameters, such as wettability and chemistry, so as to better mimic some of the intrinsic properties of the extracellular matrix (ECM). The final outcome may sometimes exert sufficient influence over the stem cells differentiation behaviours without an external growth stimulant or differentiation factors. Interestingly, it was also reported by Jonas *et al.* that culturing homotypic stem cells on such a regulated patterned surface may condition the cells differentiation towards displaying a more homogeneous cell-line as compared with those conjugated with growth factors and with a suspension culture method.^35^ In fact, controlling the size and shape of adhered stem cells were believed to offer better control of cellular attachment and spreading and subsequently differentiation.^36^ Another noteworthy mention would be the use of a co-culture system to influence cellular differentiation.^37,38^ However, this system was not as much appreciated due to difficulties with the experimental design, which might lead to a misinterpretation of the results. A micro-fabricated co-culture system was introduced by Bhatia in 1990s, but also failed to gain widespread application due to the limited choices of self-assembled molecules, leading to the formation of a less organized patterned surface.^39^ Nonetheless, the first approach for developing a co-culture *via* patterned SAMs by Muni *et al.* in 2014 could successfully mimic the *in vivo* cellular architecture and effectively exhibited cellular differentiation.^40^ By performing a co-culture of multiple cell types on a patterned SAM, the interaction between cells allowed the appropriate cell signalling to be switched and ultimately promoted the differentiation of cells in a collective fashion, although discerning their individual influences, at times, proved daunting.

Of the many stem cell types, mesenchymal stem cells (MSCs) are one of the more important and most studied cell lineage for an important reason. The control of differentiation towards useful osteoblast and chondrocytes has many important healthcare applications.^41–43^ However, the direct deliverance of MSCs to the body is not desirable due to opportunistic ‘run-away’ differentiation events, which may be more disastrous than helpful.^44,45^ Hence, the need for a controlled fashion of administering MSCs into the body has often been an important focus in the literature and external materials in the forms of biomedical implants are often used to help supplement the administration process. Over time, this subsequently resulted in the growing need to better understand the interaction mechanisms between tailored surfaces and MSCs.

From an academic point of view, any discussion of cell–surface interactions would be made simpler if only one single parameter at any time point is considered, although this may not be easily identifiable in literature due to the inclination towards more complex system designs. In truth, using a single functional group in SAMs alone could also achieve effective control over the differentiation of stem cells, but the reports of such are few and far between. Certainly, such systems would facilitate an easier examination and scrutiny of distinct surface chemical variants influence on the differentiation of stem cells. Hence, this provides the main impetus for this review, although it is important to mention that we have deliberately tried to pay special attention to SAM systems that are typically composed of a thin monolayer only exhibiting one single functional group, although this was not easy at times. In the interest of identifying how functional groups can interact with differentiation, we tried to separate polymeric systems (soft materials) whenever possible, as polymeric systems often present additional 3D structures that may sometimes act on the differentiation process. We also decided to dedicate our attention towards MSCs in view of their significance in the literature. The discussion on some of the more commonly reported functional groups on surfaces found to be able to induce stem cell differentiation is also summarized. This review will be presented in the following fashion: the discussion commences by first giving a brief introduction to SAMs and their properties, followed by the common mechanism of triggering MSC differentiation. The final bulk of this report is then focused on the different functional groups on surfaces that promote stem cells differentiation.

## 2. Self-assembled monolayers (SAMs)

SAMs are currently at the forefront of surface chemistry research and many important applications have found their origins in their development. Well-designed surface SAMs with nanoscale thickness are often described as stable, with a high order of organization with closely packed surface moieties that present highly attractive and homogenous chemistries on the surface^46^ towards incoming adherent cells. SAMs are typically amphiphilic molecules that can assemble by spontaneous adsorption or by covalent grafting onto a surface, with the intention of the formation of monolayers of controlled thickness.^47–49^ Amphiphilic molecules carry a “head group” at one end that has a special conjugation affinity towards a substrate and another terminal functional group at the distal “tail” end. It is this “tail” functional group that could be modified and is thus deliberately selected to improve the hydrophilic and hydrophobic properties of the substrate, depending on the demand applications, such as for adhesion, lubrication, wettability and protein adsorption. A well-decorated surface would enable uniform cell adhesion, growth and subsequently differentiation.^50^ For example, SAMs that are terminated with hydrophilic amine, carboxyl or epoxy groups were found to promote adsorption of the extracellular matrix (ECM) protein, which improved cellular adhesion;^51,52^ whereas poly(ethylene)glycol (PEG)-terminated monolayers, which have anti-fouling properties, effectively resist protein adsorption^53^ due to their intrinsic ability to retain a very thin layer of water.

MSCs will respond differently to diverse surface wettability profiles, which is, in turn, dependent on the terminated functional groups in the monolayer. Apart from the wide array of chemical modification strategies available, the growth of SAMs on the surfaces may also be aided in tandem by other topography defining techniques, such as photolithography, soft lithography, jet patterning and stencil-assisted patterning, as reviewed by Falconnet *et al.*^54^ Generally, a true SAM system that comprises a thin film of homogenous surface chemistry would enable a closer examination of the stem cell fate^55^ than is typically offered from surfaces grafted with polymeric-based systems, as their long-chain carbons sometimes undergo certain conformational rearrangements in response to environment variations or external stimuli as mentioned earlier. In brief, the important factors in SAMs that can affect substrate surface wettability could broadly be classified by: (1) the type of terminated functional group and (2) the chain length surface molecules. By engineering newer surface chemistries with different functional groups, this may generate a variety of interesting surface properties that could influence the conformation of the adsorbed ECM proteins^56,57^ as well as the size and shape of cells.^36,58^ So far, methyl, amine-, hydroxyl- and carboxyl-terminated SAMs have been widely used to study their influence on MSC behaviours.

## 3. Mechanism of triggering surface induced stem cell differentiation

Certainly, the different surface chemistries from SAM systems have been widely reported to possess the ability to direct cellular adhesion and it is this tailoring of the surface adhesion that would in turn alter the characteristics of the focal adhesion points and its subsequent downstream intracellular signalling cascade, which could further influence the commitment of the stem cells to the desired cell lineage. Prior to indulging immediately the various chemistry functional groups directing stem cell differentiation, it may be necessary to first discuss some of the underlying factors and background mechanisms governing the surface-induced differentiation event.

The influences from the surface chemical groups on protein adsorption can affect the overall integrin-binding profiles, which would then affect the cell adhesion, morphology and subsequent differentiation. So far, studies have indicated that integrin clustering and the formation of focal adhesions are controlled by the surface features *in vitro*, and subsequent changes in both focal adhesion density as well as the cell length may be interconnected with stem cell lineage commitment.^59–61^ The role of surface chemistry has been suggested for modulating the conformation of adsorbed ECM proteins, and the subsequent activities of the various integrin subunits. This initial stage of focal adhesion formation and intracellular signalling cascade could affect gene expression and ultimately direct stem cell fates solely from the integrin's microarchitecture and expression.^60^ Generally, the phosphorylation of focal adhesion kinase (FAK) induced by integrin α_5_β_1_ contributes towards the downstream activation of the ERK1/2 and PI3K/AKT pathways, which in turn promotes osteogenic differentiation and matrix mineralization in MSCs.^62–64^ On the contrary, the overexpression of α_v_β_3_ integrin has been suggested to have an inhibitory effect towards pro-differentiation signals triggered by α_5_β_1_ integrin, resulting in the absence of osteogenic differentiation. The expression of integrin subunits on different functional-group-terminated surfaces is summarized in Table 1 below.

**Table 1:** Expression of integrin subunits on different functional-group-terminated surfaces

Functional groups	Integrin	Outcomes	References
Methyl group, CH_3_	None	Maintain undifferentiated/quiescent state	65 and 66
Amine group, NH_2_	α_5_, α_v_ and β_1_	Osteogenic differentiation	65–67
Carboxyl group, COOH	α_5_, β_1_, α_v_ and β_3_	Chrondogenic differentiation	65 and 67
Hydroxyl group, OH	α_5_, α_v_ and β_1_	Osteogenic and chrondogenic differentiation	66, 68–70
Phosphate group	α_v_ and β_1_	Osteogenic differentiation	66

Additionally, the roles of certain signalling pathways important in MSC differentiation should also be mentioned, for example, the Wnt signalling pathway. Several reports have shown that 19 members of the Wnt family could induce two important signalling pathways, namely the canonical and non-canonical signalling pathways.^71^ Canonical Wnt signals bind β-catenin, whereas non-canonical Wnt signalling does not require β-catenin.^72^ The overexpress of Wnt/β-catenin has been correlated with the induction of osteogenesis in MSCs^73,74^ on surfaces with an appropriate surface roughness and wettability profile. BMP/Smad is another important pathway that regulates the differentiation of MSCs through the activation of different downstream pathways that arise *via* the interaction with surface-bound peptides.^75,76^ Surface-bound BMP may induce two signalling transduction pathways, namely the Smad-dependent and -independent pathways.^71^ In the Smad-independent pathway, the upregulation of FGFR3 promotes BMP-mediated chondrogenesis, while Smad-dependent signalling stimulates the osteogenesis of MSCs.^77^ Nandini *et al.* showed that PI3K/AKT was involved in Smad-dependent BMP-2 transcription and led to osteoblast differentiation,^77^ hence rendering PI3K/AKT pathway also as one of the central pathways in MSC differentiation.^78^

It is also important to mention that the lineage commitment of stem cells driven by geometric cues was also reported for smaller area being favoured for mechanically induced adipogenesis.^79,80^ Geometrically defined surface features may dictate the degree of cells spreading and the overall cytoskeletal tension^81^ as well as the Rho/ROCK pathway, which modulates switching between adipogenesis and osteogenesis. To date, RhoA, actomyosin^82^ and YAP/TAZ^83^ appear to be the molecular switches that could sense and give feedback on the mechanical forces exerted from the microenvironment and thus could subsequently direct the downstream biochemical signal transductions leading to MSC differentiation. The shape of attachment and “spreadiness” of seeded stem cells has been reported to regulate expression. This would suggest that any increment in cytoskeleton contractility would exhibit strong preferences towards the osteogenesis of MSCs. In conjunction, activated RhoA would also trigger the transcription of Runt-related transcription factor 2 (RUNX2) and ultimately induce the matrix mineralization level and differentiation of MSCs towards the osteogenic lineage. By contrast, low RhoA activity would be associated with a low cytoskeleton contractility; hence promoting adipogenesis on the cells adhering to the surface features to restrict the degree of cell spreading. From the information above, one could easily correlate how the surface chemistries are useful to dictate the overall cell surface morphology and this could in turn stimulate differentiation towards the various lineages under the context of surface-induced morphogenesis.

## 4. Coating strategies of different monolayers on surface for the modulation of stem cells

**Scheme 1:**
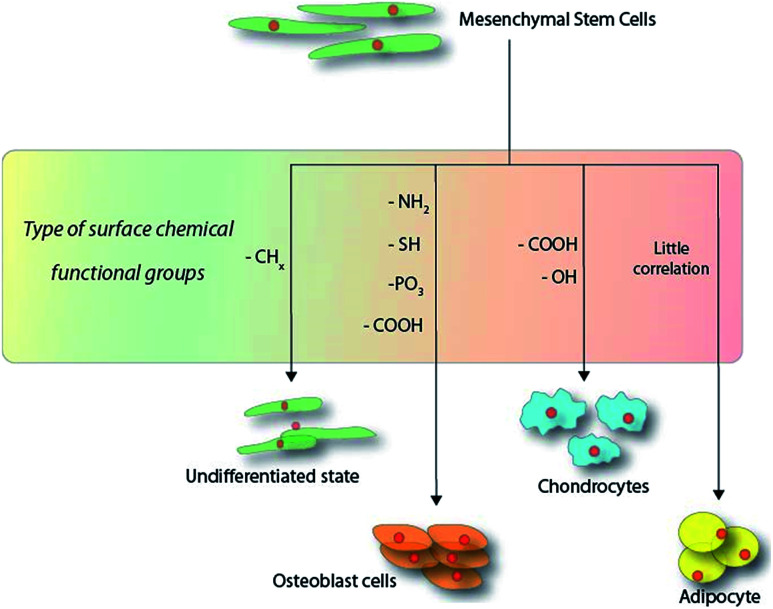
Schematic overview of the functional groups directing the outcome of mesenchymal stem cell differentiation within the first 14 days on hard substrates.

The strategies for producing organic monolayers on surfaces are highly dependent on the substrate in question and the approach may range from simple radical-based reactions^84^ to complex “click” chemistry models.^85^ It is also possible to find mentions of mechanistic strategies involving chemical vapour deposition (CVD) for means producing a thin film on surfaces, where the term “SAM” was loosely coined. Due to the wide variety of approaches, only a few notable methodologies will be discussed in the following paragraph. Scheme 1 illustrates the current observable trend observed in the surface-chemistry-induced differentiation of MSCs within the first 14 days of culture.

Typically for any given substrate that can present a free OH groups on the surface, some of the more popular interactions with silanes^86–88^ or catechol-based chemistry^89^ have often been proposed, although the need for surface hydrolysis in the physiological environment may offset its usefulness. Nonetheless, it is easy to find reports in the literature describing silanization on a wide range of surfaces, ranging from glass to even hydroxyapatite.^87^ Upon mentioning silanes, it is important to note that the cross-linking effects among the silane molecules would, in actuality, present distal functional groups that are often considered insufficiently dense enough on the surface for many researchers. Hence many would opt to take a more cautious tone when describing it as a SAM in recent years. On the question of surface packing density, another fundamental question that needs to be addressed would be definition of “closeness”. Spatz's group argued on the notion that a minimal spatial distance of 63 nm of surface ligand placement would be required for the proper formation of focal adhesion points from MSC adhering cells^90^ although this phenomenon has not yet been established as the clear order of the day. Certainly, if the distance of the focal adhesion points were to garner more weight towards the final differentiation outcome, it would be clear in the future that this spatial distancing would be the limelight in many future research studies. In terms of the grafting approach, CVD deposition remains an attractive strategy towards passivating surfaces with bioactive chemical moieties^91,92^ but often, the need for a high temperatures may render it unsuitable for many polymeric-based substrates. Many of the determining factors for choosing a chemical approach are often dependent on the biomaterial substrate in question. However, for the purpose of this review, the authors do not feel that physically absorbed molecules can be efficiently branded as SAMs due to their inherent instability in physiological environment.

The choice of substrate can also influence much of the passivation chemistry strategy that is adopted. Apart from the nominal bioactive polystyrene or glass surfaces, many authors often suggest the use of a gold surface, expressively using thiols to produce high-quality SAMs to interface with the MSCs.^93^ This is in part due to the rich dense monolayers that can be produced on gold surfaces *via* the thiolation chemistry, although certain authors may question the use of gold in stem cell differentiation due to its inability for subsequent degradation. Nonetheless, on the question of SAMs, gold remains as a very useful tool to directly examine the effects of surface chemistries. Focusing on MSC, one can also find many composite materials, such as ceramics and hydroxyapatite, proposed as viable templates for interfacing with MSCs, and many of these reports use surface chemistry modification in tandem to produce the proper desired differentiated linage. Regardless of the substrate used, many useful tools, such as FTIR, AFM, XPS, solid-state NMR, RAMAN, TOF-SIMS and STM, are readily available to systematically catalogue the composition of the surface chemistry. Nonetheless, pertaining to the various chemistry approaches available to produce SAMs on surfaces, it is in the author's opinion that this is not within the scope of this review and it is already widely covered in the literature.^89^

**Fig. 1:**
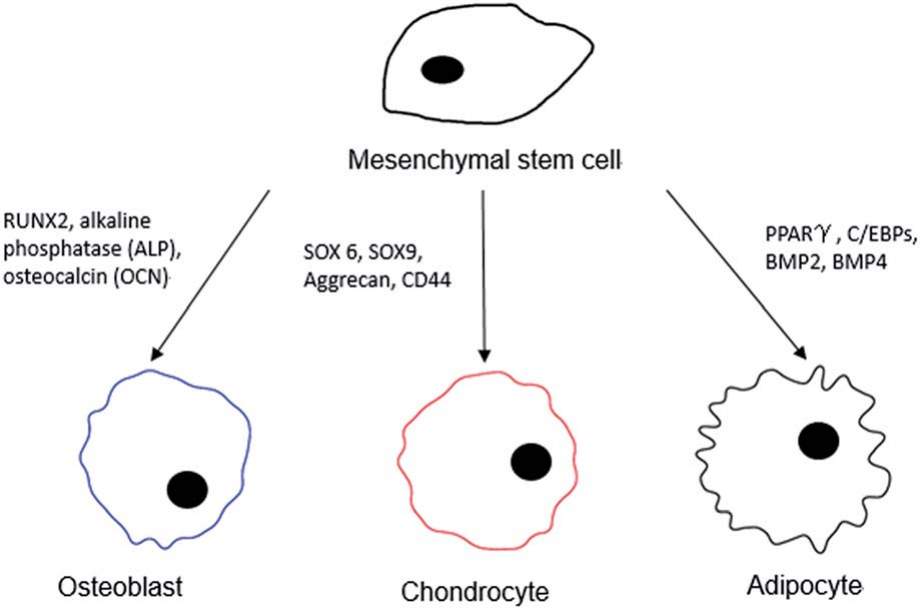
Possible surface markers and protein expression targets for the differentiated cell lineage from an MSC.

In order to better interpret the relationship between the stem cells and the different surface chemistries used in the differentiation process, a distinction should be made for a “pure” monolayer and one that is of a mixed nature. For the intended purposes of this review, we consider pure monolayers as SAMs on surfaces that display only a single chemical functional moiety at the distal end of the surface, while mixed surfaces present more than one chemical functional group during the interaction with MSCs. In the process of examining differentiation outcomes, a series of different surface markers are used to catalogue the cells, both visually and quantitatively, and these are as shown below in Fig. 1.

### 4.1 Single monolayer systems

As mentioned, the easiest way to define the relationship between surface chemistry and MSC differentiation would be using the homogenously presented chemistries on the surfaces of a single functional group. By studying the effects from single functional groups, many other parameters, such as topography and surface tension, can be initially disregarded, and hence such an approach has found much support in the literature. Below are some of the more common functional groups that have been examined currently in the community working in this field (Table 2).

**Table 2:** Some examples of SAM systems grafting with different functional groups and the outcomes

Title of publication	SAM fabrication	Functional group	Culture period	Outcomes	Year
Integrin-binding specificity regulates biomaterial surface chemistry effects on cell differentiation^62^	Gold-coated substrates	CH_3_	7 and 14 days	OH and NH_2_ upregulate osteoblast-specific gene expression compared with the COOH and CH_3_ substrates	2005
		OH			
		COOH			
		NH_2_			
The guidance of human mesenchymal stem cell differentiation *in vitro* by controlled modifications to the cell substrate^69^	Glass coverslips	CH_3_	14 days onwards	MSC phenotype unchanged	2006
		NH_2_		Promoted and maintained osteogenesis	
		SH			
		OH		Promoted and maintained chondrogenesis	
		COOH			
Human mesenchymal stem cell differentiation on self-assembled monolayers presenting different surface chemistries^65^	Gold-coated substrates	CH_3_	7, 10, 12 and 15 days (different experimental methods)	Formation of 3D cell aggregates	2010
		COOH		Displayed a cobblestone phenotype typical of osteoblasts	
		NH_2_		Promoted osteogenic differentiation	
		OH		Lower cell number	
Directing the fate of human and mouse mesenchymal stem cells by hydroxyl-methyl mixed self-assembled monolayers with varying wettability^94^	Gold slides	Mixed OH and CH_3_	7 days	Promoted the expression of α_v_β_1_ integrin of MSC	2014
				Promoted osteogenic differentiation in the presence of biological stimuli	
Effects of functional groups on the structure, physicochemical and biological properties of mesoporous bioactive glass scaffolds^95^	Mesoporous bioactive glass	SH	7 and 14 days	Stimulated adhesion, proliferation and differentiation	2015
		NH_2_			
Surface chemistry from wettability and charge for the control of mesenchymal stem cell fate through self-assembled monolayers^96^	Gold slides	OEG	7 and 14 days	Not supported cell adhesion and proliferation	2016
		CH_3_			
		PO_3_H_2_		Promoted mouse MSC (mMSC) adhesion and proliferation	
		OH		NH_2_ and PO_3_H_2_ promoted mMSC osteogenic differentiation	
		NH_2_		Upregulated the mMSC expression of integrins α_v_ and β_1_	
		COOH			

#### 4.1.1 Methyl group, CH_3_

**Fig. 2:**
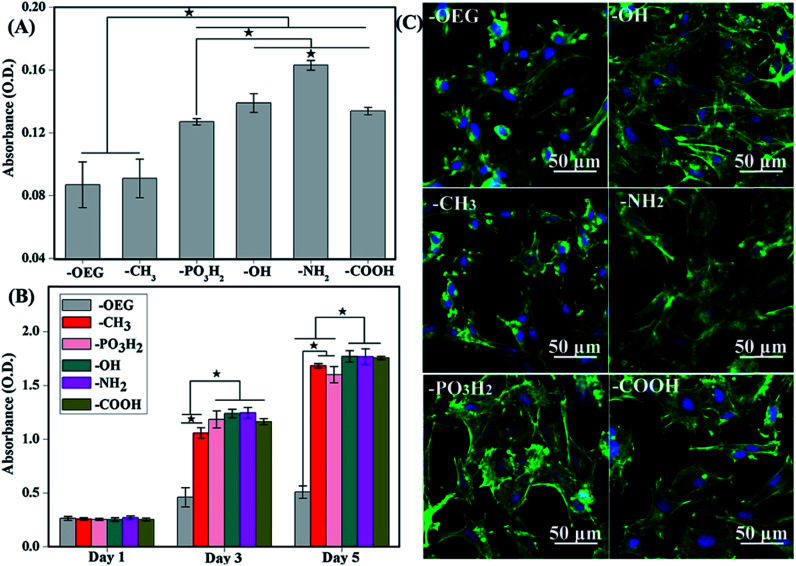
(A) Attachment of cells after 3 h of culture onto the various SAMs. (B) Proliferation of cells on SAMs of different functional groups after 1, 3 and 5 days of culture. (C) MSC morphologies after 12 h cultured on different SAMs terminated with various functional groups. Better cell spreading was observed on –OH-, –NH_2_-, PO_3_H_2_- and –COOH-terminated SAMs but not –CH_3_-terminated SAMs.^98^

Methyl group (CH_3_)-terminated SAMs have been used to prepare hydrophobic surfaces (contact angles ≥ 80°) to present a low surface energy profile to incoming MSCs.^97^ This was in an attempt to better understand how a mildly hydrophobic surface would interact with MSCs and subsequently influence the differentiation event. As expected, CH_3_-based SAMs were not found to be very bioactive and were less likely to promote MSC adhesion, hence they often maintain the undifferentiated state of the MSCs.^57^ But yet, this inactivity from the surface would actually help in the preservation of multipotency and in the self-renewal properties of MSCs, although this should be supplemented with self-renewal factors for long-term culture. The nominally observed round morphology of stem cells and the minimal spreading efficacy were due to the restriction of the surface contact areas, resulting in unfavourable cell adhesion on these hydrophobic surfaces (Fig. 2).

As observed, the formation of focal adhesion was highly regulated on the assembled fibronectin (FN). Under atomic force microscopy (AFM) examination, FN compact conformation was noticed on hydrophobic surfaces due to the rearrangement of domains that bind FN more tightly on the surface.^67,99–101^ An unusual folding of FN decreases the exposure of the binding domain, and ultimately cells would fail to induce the formation of focal adhesion, triggering the necessary downstream signalling cascade that play a role in the differentiation event.

#### 4.1.2 Amine group, NH_2_

**Fig. 3:**
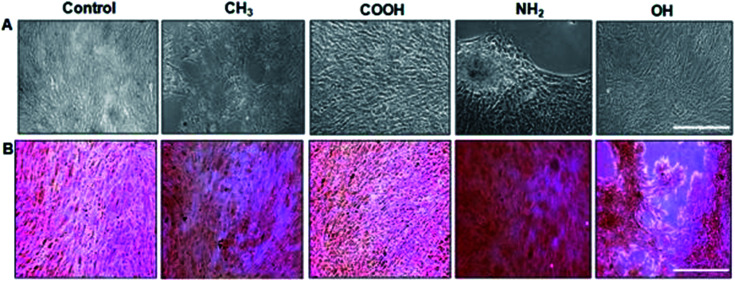
Osteoblastic differentiation: (A) morphology of unstained MSCs. Scale bar, 100 μm. (B) Matrix mineralization evaluated by Alizarin red staining for calcium deposits. NH_2_ terminated SAMs are the most favourable for osteogenesis.^65^

Amine (NH_2_)-terminated SAMs are the most commonly selected to examine the effect of positively charged hydrophilic surfaces on MSC behaviour.^66^ Multiple studies have shown that MSCs cultured in basal growth medium, in the absence of additional osteogenic supplements were found to be able to undergo osteogenic differentiation when subjected to NH_2_-functionalized surfaces (Fig. 3).^69,102,103^ From a clinical point of view, the osteogenic differentiation of MSCs is deemed crucial for the healing of large bone defects and other metabolic bone diseases. Traditionally, the *in vitro* osteogenic differentiation of MSCs was typically triggered by soluble growth factors.^104–106^ However, complications and side effects from these administrations have been reported in the literature,^107,108^ not to mention expensive synthesis costs, a short half-life, lack of specificity and ectopic tissue formation. Hence, from these aspects, SAM systems have the potential to overcome the issues associated with the solubility factors. By using different functional group-terminated SAMs, surface wettability can be improved along with the conformation of FN, hence promoting the early stage of cell spreading^109,110^ together with the eventual activation of different signalling pathways.

With NH_2_-rich surface modifications, these hydrated surfaces allow a greater conformational flexibility compared to hydrophobic surfaces, therefore permitting FN's dimer arms to extend.^111^ Extension of FN's subunits gradually increase the exposure of cryptic cell integrin-binding sequences, RGD and the PHSRN synergy domain that act towards the binding of α_5_β_1_ integrin.^112,113^ This elongated FN's conformation enables better cell–surface attachment as the augmented integrin-binding affinity quickly recruits intermediate focal adhesion components to promote further surface expansion. In conjunction, the nature of the surface charge is also known to have an important influence on cell attachment. A better cell attachment (Fig. 2) was observed on a positively charged amine surface from electrostatic interaction with the negative charges on cell surfaces.^114^ The binding of α_5_β_1_ integrin induces MSCs to undergo osteogenic differentiation *via* downstream activation of the ERK1/2 and PI3K/AKT pathways, which in turn activates the transcription of RUNX2, a critical regulator of osteogenic differentiation and matrix mineralization, as has been well established in the literature.^65,69^

#### 4.1.3 Carboxyl group, COOH

Surface charges have always been considered crucial for effective signalling of the differentiation of stem cells towards the desired lineage.^115–117^ On the other hand, carboxyl (COOH)-terminated SAMs carrying negative charges (demonstrating a modest wettability profile compared to NH_2_ SAMs) were shown to produce a markedly different conformational change in FN,^118^ resulting in the activation of a different signalling pathway that plays a role in the stem cell fate.^67^ In brief, the negative charges from COOH-SAMs cause a conformational change in FN *via* disturbing the intermolecular interactions upon its adsorption, which promotes the binding of both α_5_β_1_ and α_v_β_3_ integrins.^68^ COOH-SAMs and NH_2_-SAMs have an almost similar cell adhesion strength although they exhibit a slightly different integrin affinity as the adhesion strength is well correlated with α_5_β_1_ integrin. Instead of osteogenesis, chondrogenic differentiation is commonly observed for these negatively charged surfaces.^69^ The suppression of osteogenic phenotypes and inhibition of bone mineralization were observed on COOH-SAMs.^119^ It was shown as well that the regulation of bone mineralization was related to the β_3_ subunit as bone mineralization on COOH-SAMs was promoted when the β_3_ blocking antibody was introduced to the surface.^120^ It could be hypothesized that while the binding of α_5_β_1_ integrin promotes osteogenic differentiation, α_v_β_3_ integrin, on the other hand, supresses bone mineralization while at the same time inducing chondrogenic differentiation. These findings clearly reveal that the variation of FN conformations by surface wettability and surface charge can induce changes in the integrin-binding affinity, which subsequently activates the relatively different extracellular and intracellular signalling pathways and leads to ultimately different stem cell differentiation results.

#### 4.1.4 Hydroxyl group, OH

Holding onto the notion of surface charges, it is possible to suggest that hydroxyl (OH)-terminated SAMs create a wettability profile akin to those from amine surfaces^121^ and hence exhibit a similar FN behaviour on surfaces to those terminated by an NH_2_ group. Thus, osteogenic differentiation was shown to be also promoted on OH-terminated SAMs.^70^ However, NH_2_-terminated SAMs are frequently used in promoting osteogenic differentiation as these surfaces can offer better cell adhesion. In fact, protein adsorption profiles on the surfaces terminated with different functional groups were correlated to the cell attachment, as reported by Baugh and Vogel.^56^ It was found that OH- terminated SAMs promoted the lowest cell attachment among the hydrophilic surfaces as the cells appeared to be more rounded in shape, whereas the cells are well spread on both NH_2_- and COOH-terminated surfaces. In part, this is due to steric repulsion from a hydration layer formed on the surface, which discourages the adsorption of incoming proteins.^122^ These results clearly demonstrate that the surface chemistries affect cell behaviour with unfavourable protein adsorption. However, it does not affect the intracellular cascade triggering the stem cell towards the osteogenic lineage. On the other hand, Hao *et al.* designed a surface grafted with different ratios of –OH/CH_3_ for evaluation of the wettability effect on the MSC behaviours.^123^ In that study, a mixed surface with OH/CH_3_ (7 : 3) with a moderate wettability showed the highest expression of osteogenic markers. This indicated that not only the change of FN conformation, but also the surfaces wettability plays an important role in stem cell fate. Additionally, Curran *et al.* demonstrated that OH-SAMs actually do induce the chondrogenic differentiation of MSCs.^69^ However, this may contradict the reports by Hao *et al.* and Keselowsky *et al.*, who proposed that OH-SAMs may also promote osteogenic differentiation.

#### 4.1.5 Mercapto group, SH

So far, SAM surface functional groups had been shown to play a dominant role in MSC differentiation.^102^ However, not much work has been done with mercapto group (SH)-terminated SAMs in triggering the differentiation of MSCs. The surface wettability of SH-terminated SAMs typically falls between NH_2_- and CH_3_-terminated SAMs. Cells on these surfaces were found to be rounded in shape and the presentation of SH-SAMs showed an upregulation of collagen I synthesis but not collagen II, indicating that the chemical properties of SH-SAMs did not support chondrogenic differentiation.^69^ Immunoblotting showed significant enhanced osteogenic markers, such as ALP activity, RUNX, osteocalcin (OCN) and bone morphogenetic protein-2 (BMP-2), compared to the control, indicating activation of the differentiation pathway triggering MSC differentiation into osteoblasts.^95,102^ Furthermore, SH modified-mesoporous bioactive glass (MBG) has been shown to be favourable to human bone marrow-derived mesenchymal stem cells, as they are spindle shaped with a lot of filopodia extensions of the stem cells through SEM analysis.^95^ However, much of the molecular mechanism underlying how the SH group engages win the precise differentiation of MSCs remains unclear.

#### 4.1.6 Phosphate group, PO_3_

There are relatively few reports on how phosphate groups grafted on the surface influence the MSC responses. However, PO_3_H_2_-terminated SAM has the same wettability as NH_2_-terminated SAM and both of them are able to promote a similar bioactivation of MSCs. Hao's group showed that a PO_3_H_2_-grafted surface upregulated the level of integrins α_v_ and β_1_,^98^ which could stimulate cell adhesion, proliferation and differentiation. Furthermore, the PO_3_H_2_-terminated surface showed similar results to NH_2_- and COOH-terminated surfaces as it could promote MSC osteogenic differentiation, as darker blue-purple areas indicate better osteogenic differentiation. More studies on phosphate-terminated SAMs may be required in order to address the mechanism of MSC differentiation and the interaction between the phosphate group with stem cells, as well as the applications of these SAMs in the future.

### 4.2 Mixed surface systems

Many authors report using mixed systems due to the flexibility afforded in the confinement of MSC spatially. From earlier sections, it was found that MSC morphological conformity on surfaces can direct the final differentiation outcome. In the following section, we touch on some of the recent developments of using either mixed monolayer systems or monolayers that have been enhanced by geometrical features on the surfaces.

#### 4.2.1 Synergistic behaviour of monolayer and geometrical features

**Fig. 4:**
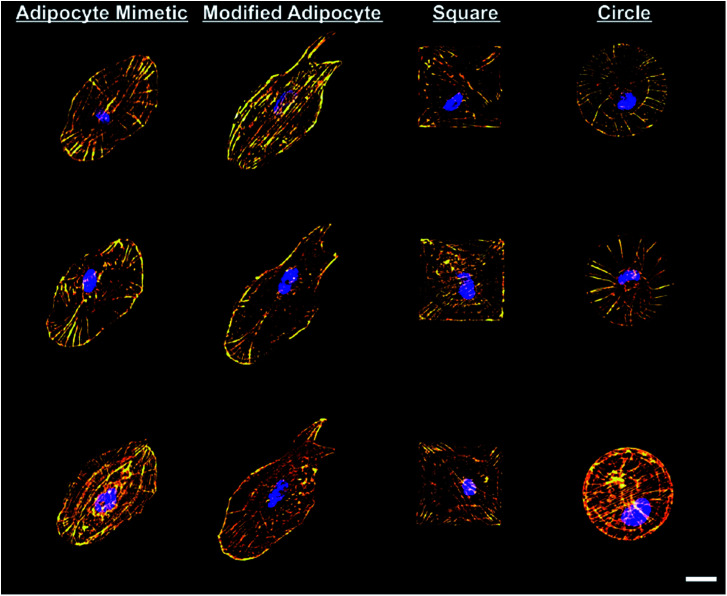
Modulation of actin cytoskeleton of stem cells on fibronectin patterns. Scale bar = 25 μm. Left to right: adipocyte mimetic, modified adipocyte, square and circle patterns. Gold = F-actin, blue = nucleus.^126^

In the past decade, geometry modulation of the cytoskeleton has been proposed as a key regulator in stem cell fate.^81^ To explain cell responses to size and shape, the microcontact printing technique has been applied with mixed SAMs on the surfaces, and multiple studies have revealed that MSCs are prone to differentiate towards the osteogenic lineage when cells are introduced in the shapes that promote the extension of the cytoskeleton.^79,124^ In addition, adipogenic differentiation was promoted when MSCs were seeded in a shape that restricts cell spreading; for example, in a circularly confined space. In a study by Kilian and colleagues, extension of the cytoskeleton was highly restricted when cells were cultured in a shape with a large convex curve.^79^ Reduction of the cell spreading consequently inhibits components in the Rho pathway, leading to the activation of PPARγ, which results in adipogenesis.^61^ A study done by Sordella *et al.* also showed the degree of cross-talking between RhoA and IGF-1 in mediating adipogenic differentiation.^125^ This study further concluded that immobilizing growth factors on surfaces may be necessary in order to induce the differentiation of MSCs towards the desired lineage. On the contrary, the gene profile showed a significant upregulation of ROCK when the cells were allowed to spread as they increased actin-myosin tension, resulting in osteogenesis. Instead of a plane geometry, cell-derived geometries were also developed by Shukla *et al. via* laser scanning lithography.^126^ Remarkably, cells residing in an adipocyte-derived geometry displayed a preference towards differentiating into mature adipocytes as compared to when in a circular geometry (Fig. 4).^126^ Such results may strongly suggest that the biomimetic geometry can enhance MSC differentiation towards the desired lineage. Thus, in tandem with the geometrical features, a synergizing approach with surface chemistry can tailor a variety of cytoskeleton extensions and hence drive differentiation to the desired cell lineage.

Muni *et al.* and Occhetta *et al.* also suggested that the secreted cellular paracrine factors can affect neighbouring cells in terms of the stimulation or repression of phenotypes that are necessary for triggering cellular differentiation.^40,127^ On the basis of this, co-culture techniques based on the use of mixed SAM systems have been utilized. These rely on the immobilization of dual self-assembled molecules to monitor the position of seeded cells as it can be easily patterned *via* photolithography and soft lithography and allows control of both the surface chemistry and the cellular environment. Furthermore, by using micro-patterned SAMs or even a 3D designed co-culture system, researchers can mimic the *in vivo* microenvironment of a heterogeneous cell population type, which would, in principle, be favourable due to the fact that cells are dependent on homotypic and heterotypic interaction^128^ for functioning or even differentiation.

#### 4.2.2 Peptide-derived SAM driving differentiation

It has been well established that collagen I plays an important role in promoting the differentiation of MSCs towards the osteogenic lineage.^129–131^ Hence, the signalling mechanism of collagen-induced osteogenic differentiation cross-talk between collagen I and the integrin (α_1_β_1_ and α_2_β_1_), which ultimately activates the downstream ERK signalling pathway, has been proposed by several researchers.^132–135^ However, the use of collagen for surface grafting was non-ideal due to undesired immunogenicity in some instances which ultimately restricts the wider application in stem cell regeneration. Furthermore, other types of xenografted proteins might carry the potential for pathogens transmission to recipients that could complicated the outcome. Hence, short peptides were suggested instead to overcome the above-mentioned issues. Additionally, the use of a peptide simplifies the examination process due to their designated interaction with solely single integrin components.

The influence of collagen-based mimetic peptides on MSC differentiation was reported by Hennessy and co-workers in 2009.^136^ In that particular study, three collagen mimetic peptides, namely DGEA, P15 and GFOGER, were grafted on the surface for evaluation of the potential of MSC differentiation towards the osteogenic lineage. The upregulation of osteogenic markers for surfaces grafted with DGEA and P15 was observed in the absence of differentiation-inducing supplements. Another experimental design for studying the influence of ECM mimetic peptides towards MSC differentiation was done by Anderson *et al.*^137^ MSCs were seeded onto the RGDS-, DGEA- and KRSR-grafted surfaces and successful osteogenic differentiation was evaluated by osteogenic markers and mineral deposition. Among the three surfaces, the RGDS-grafted surface was found to promote the highest osteogenic differentiation level, followed by the DGEA-grafted surfaces.

**Fig. 5:**
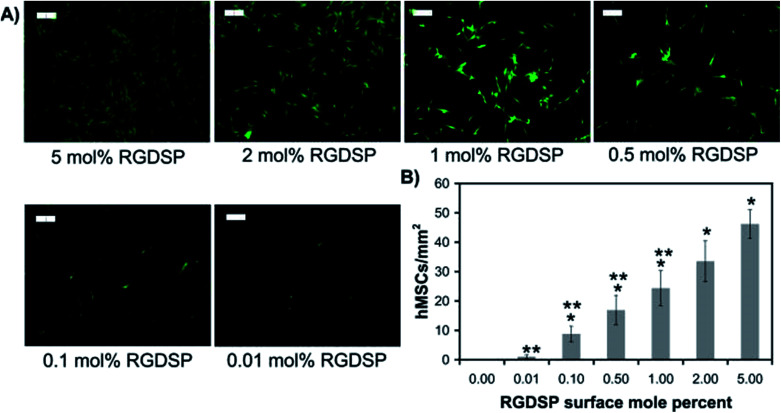
Human mesenchymal stem cell (hMSC) adhesion onto RGDSP-presenting SAMs. (A) Fluorescent photomicrographs of MSC stained with Calcein AM 24 h after seeding. (B) The attachment of hMSC per square millimetre under the RGDSP surface.^139^

In 1998, Roberts *et al.* began describing a SAM system with tripeptide, arginine-glycine-aspartate (RGD), which could stimulate the adhesion of cells through binding to cell surface integrin receptors.^138^ Stem cells behaviour, including cell adhesion, spreading and focal adhesion complex formation, promoted *via* the mixed SAM system play encouraging roles in differentiation. Murphy *et al.* also prepared a mixed SAM peptide Arg-Gly-Asp-Ser-Pro (RGDSP) by using a Cu(i)-catalyzed azide–alkyne cycloaddition (CuAAC) “click” reaction.^139^ They found that the number of human MSCs attached on the RGDSP surface was dependent on the concentration of RGDSP (Fig. 5). The results demonstrated that for the RGDSP surface, the mixed SAM could promote human MSC adhesion, spreading and the formation of cell focal adhesion. However, the outcome of differentiation from the effects of RGD remain relatively vague, especially with regards to the nature of the substrate at hand. For instance, Re'em *et al.* were able to report the promotion of chondrocytes on RGD grafted on a macroporous alginate-based scaffold^75^ while Cao *et al.* showed that cyclic RGD may promote osteoblast formation on a flat quartz substrate, attributed to the surface charges as presented.^140^ Wang *et al.* further demonstrated that RGD could be used to promote osteogenesis on to deliberate gold nanopatterns,^141^ hence demonstrating the complexity in examining and cataloguing differentiation trends if parameters such as the geometrical features are factored in.

Interestingly, the immobilization of growth factors with the SAM approach has shown them as excellent mediators for differentiations. Park *et al.* immobilized bone morphogenesis protein (BMP-2) on carbon nanotubes and the material was found to drive osteogenesis and chondrogenesis.^142^ Similarly, Schwab *et al.* demonstrated that immobilized BMP-2 proteins through SAM fashion were able to stimulate the precursors of osteogenesis from MSCs^76^ and argued that the immobilized BMP-2 were more efficient in triggering osteogenesis. Mao *et al.* further claimed that immobilized growth factors were indeed more stable with a longer shelf-life compared to the soluble forms.^143^ Considering that this approach has yet to attain the point of wider application, it is impossible to state categorically that the immobilization of grow factors can better serve to deliver bioactivity than when in the solubilized form, although the trend seemingly suggests so.

### 4.3 “Soft” substrate *vs.* “hard” substrates

So far, the discussion in this paper has been predominantly focused on “hard” substrates, such as gold, glass coverslips and other metallic surfaces. This was deliberately done in view of the purpose of producing a highly controlled “self-assembly” chemistry. With “softer” substrates, such as polymeric hydrogels, to attempt the direct correlation between the chemical functional groups and MSC differentiation is in principle challenging. However, it is in our interest that a brief discussion should be made in order to provide a more holistic picture.

Of the various surface characteristics, surface “stiffness” has often been considered as a principle parameter by many research groups as a key element for the differentiation of MSCs.^144–146^ For example, Engler *et al.* made an important study of the surface stiffness on polyacrylamide gels and stated that softer substrates (0.1–1 kPa) lead to the formation of neurons, while MSCs grown on harder substrates (25–40 kPa) result in osteogenesis.^147^ Trappmann *et al.* described the precise ECM feedback mechanism engaged by MSCs when they come into contact with surfaces of various stiffnesses and this has a direct effect on the overall differentiation outcome.^148^ However, solely relying on the surface stiffness to drive MSC differentiation was not absolute as the surface chemical functionality may also drive various differentiation patterns of MSCs. On a soft polyethylene glycol-based hydrogel substrate grafted with various functional groups, Benoit *et al.* were able to show the differentiation to osteoblast *via* charged phosphate groups, while COOH^−^ groups help induce chondrogenesis.^149^ From the authors point of view, the synergistic relationship between the surface stiffness and chemistry presented from self-assembled monolayers still remains one important and yet overlooked aspect of MSC differentiation. However, with “softer” substrates, the chemistry presentation of the material (typically polymer based) to the adhering MSCs is often rich and heterogeneous and this renders an immediate correlation between surface chemistry and MSC difficult, and for this reason, herein, the authors decided to focus predominantly on hard surfaces in this review.

## 5. Conclusions

The objective of this review was not only to understand the mechanism of triggering stem cell differentiation, but also to explore the interaction between surface chemistry and MSC differentiation. The natural interaction between cells with the grafting surface is a complex bi-directionally process. In this review, the differentiation lineage and its correlation with surface chemistry have been mapped and an important trend could be seen emerging from the information presented. It is obviously clear that cell adhesion plays an important role and how well cells adhere to the surface can drive its differentiation outcome. Interestingly, we noticed that there were fewer correlations between the surface chemistry and the adipogenesis of MSCs on hard substrates. Instead, surface topography was noticed to play a more important role for adipogenesis compared to the surface chemistry.

In summary, it was observed in the literature that different types of monolayers coating surface can modulate the stem cells' fate even in the absence of external chemical stimulants such as cytokines. OH-, COOH- and NH_2_-terminated surfaces were found to promote the adhesion, proliferation and differentiation of MSCs. Another important feature would be mixed monolayers system, by which two or more functional groups are grafted collectively onto a surface, as well as SAM-presenting bioactive peptides/growth factors, which could further improve MSC adhesion and stimulate signal pathways for differentiation.

So far, we also identified various interesting areas that could have much potential for future studies. First, a quick search in the literature did not yield any notable publications pertaining to the use of SAM systems to dedifferentiate stem cells and although we suspect that the exerting factors from the surface may not be sufficient to dedifferentiate MSCs, a single report by Tan *et al.* demonstrating the dedifferentiation of meniscal cells from surface chemistry may present an interesting proposition for MSCs.^150^ Certainly, in view of the linkage between embryonic stem cells and MSCs, there might be an avenue for surface-modified functional groups interplay between both cell types, although as mentioned earlier, the differentiation of stem cells involves a series of complicated events that cannot be accounted for by one single parameter, namely from the surface. On the basis of this presentation of information, the work on SAMs stimulating MSC differentiation continues to possess the potential to replace or complement conventional soluble bioactive cues as a viable alternative in stem cell regeneration technologies. This is in view of the challenges faced when trying to attain a complete homogeneous differentiation of MSCs. Thus, a more realistic scenario that the authors are suggesting here would be that surface chemistries should be collectively considered as an important parameter when attempting the differentiation of MSCs to a specific cell type, especially in conjunction with growth factors and other supplements. In conclusion, the authors believe that the information in this review should be able to provide a clearer picture of how surface chemistries can influence MSC differentiation outcomes.

## Conflicts of interest

There are no conflicts to declare.

## Supplementary Material
